# Prognostic impact of Ki‐67 proliferative index in resectable pancreatic ductal adenocarcinoma

**DOI:** 10.1002/bjs5.50175

**Published:** 2019-05-10

**Authors:** I. Pergolini, S. Crippa, M. Pagnanelli, G. Belfiori, A. Pucci, S. Partelli, C. Rubini, P. Castelli, G. Zamboni, M. Falconi

**Affiliations:** ^1^ Department of Surgery Università Politecnica delle Marche Ospedali Riuniti, Ancona Italy; ^2^ Department of Pathology Università Politecnica delle Marche Ospedali Riuniti, Ancona Italy; ^3^ Pancreatic Surgery Unit, Pancreas Translational and Clinical Research Center, Università Vita e Salute IRCCS San Raffaele Scientific Institute Milan Italy; ^4^ Department of Pathology Ospedale Sacro Cuore – Don Calabria Negrar Italy; ^5^ Department of Pathology Università di Verona Verona Italy

## Abstract

**Background:**

Pancreatic ductal adenocarcinoma (PDAC) is an aggressive disease characterized by complex biological features and poor prognosis. A prognostic stratification of PDAC would help to improve patient management. The aim of this study was to analyse the expression of Ki‐67 in relation to prognosis in a cohort of patients with PDAC who had surgical treatment.

**Methods:**

Patients who had pancreatic resection between August 2010 and October 2014 for PDAC at two Italian centres were reviewed retrospectively. Patients with metastatic or locally advanced disease, those who received neoadjuvant chemotherapy, patients with PDAC arising from intraductal papillary mucinous neoplasm and those with missing data were excluded. Clinical and pathological data were retrieved and analysed. Ki‐67 expression was evaluated using immunohistochemistry and patients were stratified into three subgroups. Survival analyses were performed for disease‐free (DFS) and disease‐specific (DSS) survival outcomes according to Ki‐67 expression and tumour grading.

**Results:**

A total of 170 patients met the selection criteria. Ki‐67 expression of 10 per cent or less, 11–50 per cent and more than 50 per cent significantly correlated with DFS and DSS outcomes (*P* = 0·016 and *P* = 0·002 respectively). Ki‐67 index was an independent predictor of poor DFS (hazard ratio (HR) 0·52, 95 per cent c.i. 0·29 to 0·91; *P* = 0·022) and DSS (HR 0·53, 0·31 to 0·91; *P* = 0·022). Moreover, Ki‐67 index correlated strongly with tumour grade (*P* < 0·001). Patients with PDAC classified as a G3 tumour with a Ki‐67 index above 50 per cent had poor survival outcomes compared with other patients (*P* < 0·001 for both DFS and DSS).

**Conclusion:**

Ki‐67 index could be of use in predicting the survival of patients with PDAC. Further investigation in larger cohorts is needed to validate these results.

## Abstract (es)

**Antecedentes:**

El adenocarcinoma ductal de páncreas (*pancreatic ductal adenocarcinoma*, PDAC) es una enfermedad agresiva con características biológicas complejas y pronóstico pobre. La estratificación pronóstica del PDAC ayudaría a mejorar el tratamiento del paciente. El objetivo de este estudio era analizar la expresión de Ki‐67 como marcador pronóstico en una cohorte de pacientes con PDAC tratados quirúrgicamente.

**Métodos:**

Se efectuó un análisis retrospectivo de pacientes sometidos a resección pancreática por PDAC en dos centros italianos entre agosto de 2010 y octubre de 2014. Se excluyeron los pacientes con enfermedad metastásica o localmente avanzada, los tratados con quimioterapia neoadyuvante, los pacientes con PDAC originado en una neoplasia papilar mucinosa intraductal y aquellos pacientes con datos incompletos. Se analizaron los datos clínicos y anatomopatológicos. La expresión de Ki‐67 se evaluó por inmunohistoquímica y los pacientes se estratificaron en tres grupos. Se calculó la supervivencia libre de enfermedad (*disease‐free survival*, DFS) y la supervivencia específica de la enfermedad (*disease‐specific survival*, DSS) según la expresión de Ki‐67 y el grado tumoral.

**Resultados:**

Un total de 170 pacientes cumplió los criterios de selección. La expresión de Ki‐67 del ≤ 10%, 11‐50% y > 50% mostró una correlación significativa con los resultados de DFS y DSS (*P* = 0,016 y *P* = 0,002, respectivamente). El índice Ki‐67 fue un predictor independiente de pobre DFS (cociente de riesgos instantáneos, *hazard ratio*, HR 0,52, i.c. del 95% 0,29‐0,91; *P* = 0,022) y DSS (HR 0,53, i.c. del 95% 0,31‐0,91; *P* = 0,022). Asimismo, el índice Ki‐67 se correlacionaba fuertemente con el grado tumoral (*P* < 0,001). Los pacientes con un PDAC clasificado como tumor grado G3 y con un índice Ki‐67 > 50% tenían peores resultados de supervivencia en comparación con otros pacientes (*P* < 0,001 para ambos DFS y DSS).

**Conclusión:**

El índice Ki‐67 se puede utilizar como predictor de supervivencia en pacientes con PDAC. Hace falta seguir investigando para validar estos resultados en cohortes más grandes.

## Introduction

Pancreatic ductal adenocarcinoma (PDAC) is an aggressive disease characterized by complex biological features and a poor prognosis^1^, ^2^. Recent literature^3^, ^4^, ^5^ in this field has focused on molecular biomarkers and targets to improve staging, treatment and, consequently, patient survival.

The expression of Ki‐67 in tumour tissue is a well known marker associated with tumour proliferation and correlated with the progression, risk of metastasis and prognosis of several tumours, including breast and prostate cancers^6^, ^7^, ^8^, ^9^, ^10^. In pancreatic neuroendocrine neoplasms (PanNENs), Ki‐67 has been documented to play an essential role in defining tumour grading and classification (WHO 2017/ENETS criteria), and is recognized as an independent predictor of survival^11^, ^12^, ^13^, ^14^, ^15^, ^16^. Moreover, some authors^17^, ^18^, ^19^, ^20^ have reported that the Ki‐67 index could be determined from selected PanNEN samples obtained by endoscopic ultrasonography–fine‐needle aspiration (EUS–FNA), thereby demonstrating its value in the preoperative phase. In PDAC, the prognostic value of Ki‐67 has not yet been established^21^, ^22^, ^23^. The aim of this study was to analyse the expression of Ki‐67 as a prognostic factor in a cohort of patients with resected PDAC, in relation to survival outcomes.

## Methods

This study was designed according to the REMARK^24^ and STROBE^25^ guidelines. It was not preregistered with an analysis plan in an independent institutional registry.

Patients who had a pancreatic resection for histologically confirmed PDAC between August 2010 and October 2014 at the Ospedale Sacro Cuore – Don Calabria (Negrar, Verona, Italy), a teaching hospital affiliated to the University of Verona, and at the Ospedali Riuniti Ancona Università Politecnica delle Marche (Ancona, Italy), a university hospital and referral centre for hepatobiliopancreatic surgery in the Marche Region, were reviewed retrospectively. Surgical resections were performed in both centres by the same surgeons. Both institutions were documented as high‐volume centres for pancreatic surgery (more than 100 pancreatic resections annually) at the time of the study.

Patients with metastatic or locally advanced disease, those who had received neoadjuvant chemotherapy, patients with PDAC arising from intraductal papillary mucinous neoplasms, and patients with missing data or follow‐up were excluded. Written informed consent for use of their personal data and tissue for research purposes was obtained from all patients included in the study. Institutional review board approval was not required owing to the retrospective nature of the study.

Data on patient demographics, clinical presentation, tumour marker levels (serum carbohydrate antigen (CA) 19‐9 and carcinoembryonic antigen (CEA)), preoperative treatments, surgical and postoperative data, including delivery of adjuvant treatment, were recorded. In the absence of jaundice, the preoperative concentration of CA19‐9 was recorded; in patients with abnormal serum bilirubin values at the time of diagnosis, the CA19‐9 level was determined after biliary drainage and jaundice resolution. Pathology data included tumour size and grade, number of resected/positive lymph nodes, TNM staging, lymphatic and vascular invasion, perineural invasion and margin status. Glandular differentiation and mitotic activity were evaluated in the entire tumour specimen and the more severe grades were recorded. TNM staging was done in accordance with the 7th AJCC system^26^, and margin status was determined according to the 2010 WHO definition^27^.

### Ki‐67 expression

Formalin‐fixed specimens were processed into paraffin according to standard practice. Sections (5 μm) were stained with haematoxylin and eosin for conventional histological examination, and used for Ki‐67 immunohistochemical analysis. For Ki‐67 immunohistochemical staining, after deparaffinization in xylene for 30–40 min, the specimen slides were rehydrated in a descending alcohol series, from absolute ethanol to distilled water. Before staining, in order to retrieve antigen epitopes, the samples were heated in an aqueous sodium citrate solution in a microwave oven (temperature 98°C, pH 6) for 20 min. After microwave treatment, the sections were cooled down for a further 20 min. Endogenous peroxidase was blocked by 0·3 per cent hydrogen peroxide for 7 min. After washing in Tris‐buffered saline (TBS), the slides were incubated at room temperature for 30 min with the primary antibody for Ki‐67. The primary antibody was a monoclonal mouse antihuman Ki‐67 antigen (MIB‐1; Dako, Glostrup, Denmark) used at a dilution of 1 : 80. After incubation, the primary antibody was washed away with TBS. The slides were then incubated at room temperature for 20 min, using the visualization system EnVision™ FLEX/HRP (Dako) containing the secondary antimouse/rabbit antibody. Final staining was done with diaminobenzidine tetrahydrochloride (DAB) solution for 10 min at room temperature. Slides were then transferred through an ascending ethanol series, finally through xylene, and then mounted.

Two tissue blocks for each patient were selected from the most representative area of the tumour (the region of the tumour with highest grade). A section of each block was immunolabelled for Ki‐67 using the above protocol. Counting of tumour cells was done manually using a Nikon Eclipse 80i microscope (Nikon Instruments, Amsterdam, the Netherlands), at 40× magnification. A counting protocol of 1000 cells was chosen to overcome the marked cellular heterogeneity for each carcinoma, as the number of high‐power fields could be variable. The percentage of Ki‐67‐positive cells was determined by scoring a minimum of 1000 cells within a hotspot area (defined as the area in which the 1000‐cell count provided the highest percentage of Ki‐67‐positive nuclei). Of note, the Ki‐67 index was counted in hotspot areas that did not necessarily parallel the histological grade field by field.

### Outcome measure

Primary outcome measures were disease‐free survival (DFS), the first recurrence of cancer after surgery, and disease‐specific survival (DSS), death from the disease. Follow‐up was done on a regular basis by clinical evaluation or telephone interview, and patients were censored at the last available contact date.

### Statistical analysis

Continuous variables are reported as median (range) values, and categorical variables as numbers with percentages. Continuous variables were dichotomized around the median value, except for CA19‐9, for which a cut‐off value of 200 units/ml or more was previously documented^28^, ^29^ to correlate with tumour burden, spread and early recurrence after resection of PDAC. Student's *t* test was used to compare normally distributed continuous variables; non‐parametric analyses included Mann–Whitney *U* and Kruskal–Wallis tests. Survival analysis was done with the Kaplan–Meier method and log rank test using the following Ki‐67 cut‐off values: 10, 20, 30, 40, 50 and 60 per cent, tertiles and quartiles. Patients were also stratified according to Ki‐67 index and tumour grades, and survivals were calculated accordingly.

Multivariable analysis was performed using the Cox regression model to evaluate significant predictors of DFS and DSS. Significant variables in the univariable analysis were included as co‐variables; *P* ≤ 0·050 was considered significant. Statistical analyses were performed in SPSS® version 22.0 for Windows® (IBM, Armonk, New York, USA).

## Results

Of 272 patients who underwent resection for PDAC during the study period, 170 met the selection criteria (*Fig*. S1, supporting information).

Patient characteristics, surgical and pathological data are presented in *Table* 1. PDACs were poorly differentiated (grade G3) in 40·6 per cent of patients, assessed as having T3 status in 87·1 per cent, with lymph node metastasis in 71·8 per cent of the cohort. Lymphatic invasion was documented in 100 per cent of the tumours with a positive N status, but was present in only 8 per cent (4 of 48) of N0 tumours. Some 67·6 per cent of tumours showed microvascular invasion and 85·3 per cent had perineural invasion. Stage IIb tumours were found in 72·4 per cent of patients. The median Ki‐67 index was of 30 (range 2–95) per cent.

**Table 1:** Details of patients who had upfront surgery

	No. of patients* (*n* = 170)
Age (years)†	70 (44–85)
Sex ratio (M : F)	92 : 78
Preoperative tumour marker levels†	
CEA (ng/ml)	2 (0–90)
CA19‐9 (units/ml)	36 (0–2689)
Jaundice at diagnosis	113 (66·5)
Duration of surgery (min)†	343 (120–575)
Postoperative complications	86 (50·6)
Pancreatic fistula	46 (27·1)
Biliary fistula	12 (7·1)
Duration of hospital stay (days)†	11 (5–70)
Readmission	50 (29·4)
Ki‐67 index (%)†	30 (2–95)
≤ 10	43 (25·3)
11–50	106 (62·4)
> 50	21 (12·4)
Tumour size (mm)†	25 (2–70)
Grade of differentiation	
G1	15 (8·8)
G2	86 (50·6)
G3	69 (40·6)
T category	
T1	12 (7·1)
T2	10 (5·9)
T3	148 (87·1)
T4	0 (0)
N category	
N0	48 (28·2)
N1	122 (71·8)
Resection margin	
R0	120 (70·6)
R+	50 (29·4)
Lymphatic invasion	126 (74·1)
Vascular invasion	115 (67·6)
Perineural invasion	145 (85·3)
Stage	
Ia	7 (4·1)
Ib	2 (1·2)
IIa	38 (22·4)
IIb	123 (72·4)
Adjuvant treatment	166 (97·6)
Recurrence	135 (79·4)
Died	
Yes, from other cause	6 (3·5)
Yes, from pancreatic cancer progression	109 (64·1)

*With percentages in parentheses unless indicated otherwise; †values are median (range). CEA, carcinoembryonic antigen; CA, carbohydrate antigen.

### Survival outcomes

**Figure 1:**
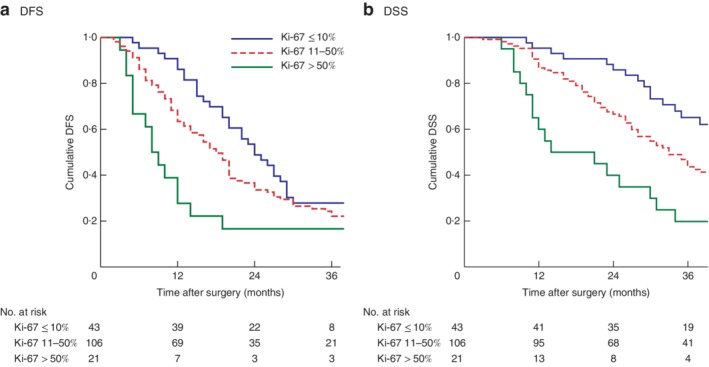
Kaplan–Meier analysis of survival in Ki‐67 index subgroups. **a** Disease‐free (DFS) and **b** disease‐specific (DSS) survival in patients with a Ki‐67 index of 10 per cent or less, 11–50 per cent and more than 50 per cent. **a** *P* = 0·016, **b** *P* = 0·002 (log rank test)

Median follow‐up was 32 (range 0–76) months. Some 135 patients (79·4 per cent) had a recurrence. Median DFS was 19 (i.q.r. 35–10) months, and median DSS was 35 (not reached to 21) months. Ki‐67 expression of 10 and 50 per cent were the only cut‐off values significantly associated with DFS and DSS. On this basis, survival analysis was determined using the following Ki‐67 intervals: 10 per cent or less, 11–50 per cent and more than 50 per cent. Median DFS was 24, 18 and 8 months for these respective Ki‐67 index values (*P* = 0·016) (*Fig*. 1 *a* and *Table* 2). Cox regression analysis showed that Ki‐67 index (hazard ratio (HR) 0·52, 95 per cent c.i. 0·29 to 0·91; *P* = 0·022), N status (HR 2·28, 1·48 to 3·53; *P* < 0·001) and resection margin status (HR 1·55, 1·06, 2·28; *P* = 0·024) were independent predictors of DFS (*Table* 2).

**Table 2:** Univariable and multivariable analyses of predictors of disease‐free survival

	Univariable analysis			Multivariable analysis	
	*n*	Median DFS (months)	*P*	Hazard ratio	*P*
Age (years)			0·101		
≤ 70	93	20			
> 70	77	18			
Sex			0·821		
M	92	19			
F	78	20			
Jaundice			0·665		
No	57	19			
Yes	113	19			
Preoperative CA19·9 (units/ml)			0·052		
≤ 200	135	17			
> 200	35	24			
Preoperative CEA (ng/ml)			0·562		
≤ 2	109	18			
> 2	61	20			
Postoperative complications			0·894		
No	84	20			
Yes	86	18			
Ki‐67 index (%)			0·016	0·52 (0·29, 0·91)	0·022
≤ 10	43	24			
11–50	106	18			
> 50	21	8			
Tumour size (mm)			0·783		
≤ 25	98	19			
> 25	72	17			
Grade of differentiation			0·039	0·77 (0·54, 1·12)	0·169
G1	15	29			
G2	86	20			
G3	69	13			
T category			0·732		
T1–2	22	20			
T3	148	19			
N category			< 0·001	2·28 (1·48, 3·53)	< 0·001
N0	48	26			
N1	122	16			
Margin status			0·026	1·55 (1·06, 2·28)	0·024
R0	120	20			
R+	50	13			
Vascular invasion			0·756		
No	55	19			
Yes	115	20			
Perineural invasion			0·452		
No	25	19			
Yes	145	19			
Stage			< 0·001		
Ia, Ib, IIa	47	29			
IIb	123	16			
Adjuvant treatment			0·842		
No	4	26			
Yes	166	19			

Values in parentheses are 95 per cent confidence intervals. DFS, disease‐free survival; CA, carbohydrate antigen; CEA, carcinoembryonic antigen.

DSS decreased significantly in the 10 per cent or less, 11–50 per cent and more than 50 per cent subgroups (47 *versus* 33 *versus* 14 months respectively; *P* = 0·002) (*Fig*. 1 *b*; *Table* 3). Cox regression analysis identified Ki‐67 index (HR 0·53, 95 per cent c.i. 0·31 to 0·91; *P* = 0·022), tumour grade (HR 0·63, 0·43 to 0·94; *P* = 0·022), N status (HR 3·37, 1·42 to 3·94; *P* = 0·001) and resection margin status (HR 1·93, 1·28 to 2·89; *P* = 0·002) as independent predictors of DSS (*Table* 3).

**Table 3:** Univariable and multivariable analyses of predictors of disease‐specific survival

	Univariable analysis			Multivariable analysis	
	*n*	Median DSS (months)	*P*	Hazard ratio	*P*
Age (years)			0·573		
≤ 70	93	33			
> 70	77	35			
Sex			0·705		
M	92	35			
F	78	34			
Jaundice			0·597		
No	57	32			
Yes	113	35			
Preoperative CA19·9 (units/ml)			0·344		
≤ 200	135	33			
> 200	35	37			
Preoperative CEA (ng/ml)			0·626		
≤ 2	109	33			
> 2	61	36			
Postoperative complications			0·918		
No	84	36			
Yes	86	34			
Ki‐67 index (%)			0·002	0·53 (0·31, 0·91)	0·022
≤ 10	43	47			
11–50	106	33			
> 50	21	14			
Tumour size (mm)			0·697		
≤ 25	98	35			
> 25	72	33			
Grade of differentiation			0·001	0·63 (0·43, 0·94)	0·022
G1	15	n.r.			
G2	86	38			
G3	69	25			
T category			0·106		
T1–2	22	56			
T3	148	33			
N category			< 0·001	3·37 (1·42, 3·94)	0·001
N0	48	n.r.			
N1	122	30			
Margin status			0·003	1·93 (1·28, 2·89)	0·002
R0	120	41			
R+	50	27			
Vascular invasion			0·337		
No	55	34			
Yes	115	35			
Perineural invasion			0·128		
No	25	66			
Yes	145	33			
Stage			< 0·001		
Ia, Ib, IIa	47	n.r.			
IIb	123	30			
Adjuvant treatment			0·406		
No	4	56			
Yes	166	34			

Values in parentheses are 95 per cent confidence intervals. DSS, disease‐specific survival; CA, carbohydrate antigen; CEA, carcinoembryonic antigen; n.r., not reached.

Stage and lymphatic invasion were not considered in the Cox regression analysis because of the overlap with N status.

### Ki‐67 and grading

**Figure 2:**
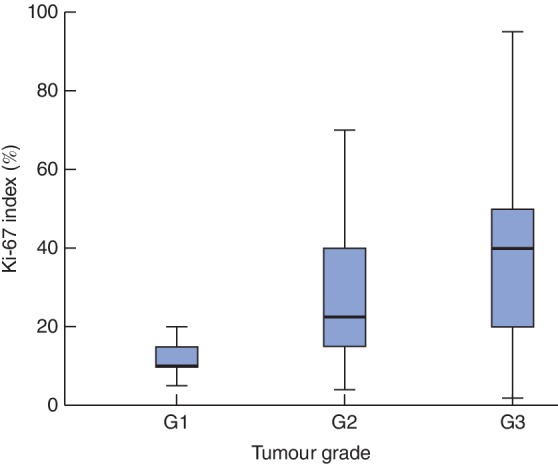
Box‐and‐whisker plot of Ki‐67 index according to tumour grade of differentiation. Median Ki‐67 index values, interquartile ranges and ranges are denoted by horizontal bars, boxes and error bars respectively. *P* < 0·001 (Kruskal–Wallis test)

Median Ki‐67 was significantly higher in G3 tumours (*Fig*. 2). Tumours with a Ki‐67 index above 50 per cent showed more aggressive grading: 62 per cent (13 of 21) had a pathological grade consistent with G3, whereas none was assessed as G1. By contrast, G3 tumours showed a more heterogeneous Ki‐67 expression (*Fig*. S2, supporting information). In patients with G3 tumours, a Ki‐67 index above 50 per cent was associated with significantly worse median survival than a Ki‐67 index of 50 per cent or less (DFS: 7 *versus* 15 months respectively, *P* = 0·035; DSS: 13 *versus* 29 months, *P* = 0·038). There was no association between Ki‐67 index and other pathological parameters, including T status, N status, tumour size, vascular or perineural invasion.

**Figure 3:**
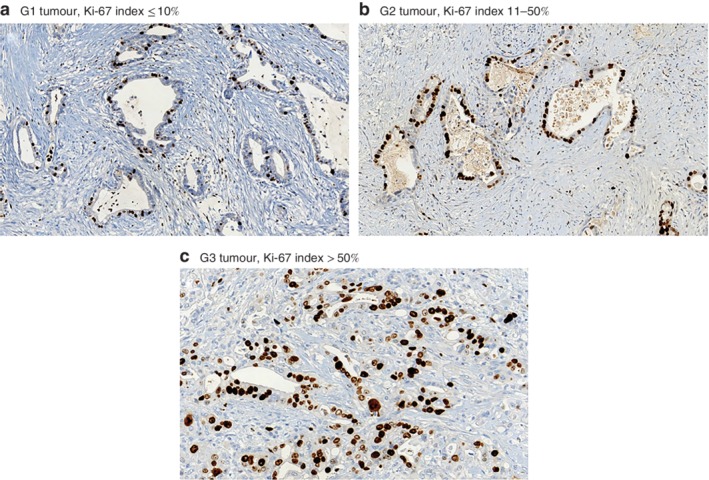
Ki‐67 immunohistochemical staining in pancreatic ductal adenocarcinoma. **a** G1 tumour with Ki‐67 index of 10 per cent or less; **b** G2 tumour with Ki‐67 index of 11–50 per cent; **c** G3 tumour with Ki‐67 index above 50 per cent

**Figure 4:**
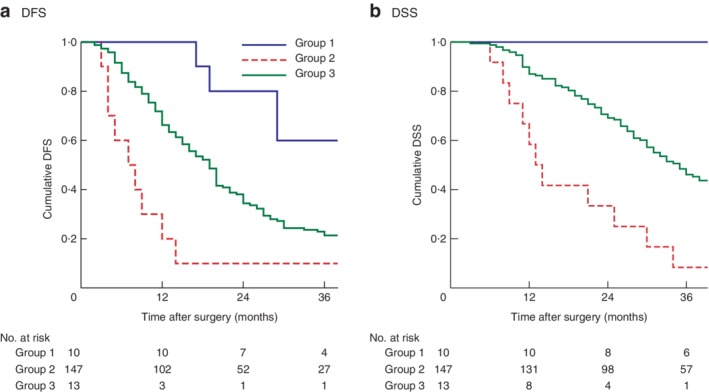
Kaplan–Meier analysis of survival according to Ki‐67 and tumour grade. **a** Disease‐free (DFS) and **b** disease‐specific (DSS) survival in patients with G1 tumours and Ki‐67 index of 10 per cent or less (group 1), G3 tumours and Ki‐67 index above 50 per cent (group 2), and all other patients (G1 tumours and Ki‐67 index above 10 per cent, G2 tumours with any Ki‐67 value and G3 tumours with Ki‐67 index of 50 per cent or less) (group 3). **a,b** *P* < 0·001 (log rank test)

Patients were categorized into three subgroups: patients with G1 tumours with a Ki‐67 index of 10 per cent or less (group 1); patients with G3 tumours with a Ki‐67 index above 50 per cent (group 2); all other patients (those with G1 tumours with a Ki‐67 index above 10 per cent, G2 tumours with any Ki‐67 index value and G3 tumours with a Ki‐67 index of 50 per cent or less) (group 3) (*Fig*. 3). Patients in group 2 had poor median survival outcomes compared with those in groups 1 and 3 (DFS: 7 months *versus* median survival not reached *versus* 19 months respectively, *P* < 0·001; DSS: 13 months *versus* median survival not reached *versus* 35 months, *P* < 0·001) (*Fig*. 4).

## Discussion

Surgical resection followed by adjuvant chemotherapy/chemoradiotherapy is considered the standard of care for localized and resectable pancreatic cancer; however, the majority of patients develop tumour recurrence, and up to 30 per cent die within 1 year after surgery^29^, ^30^, ^31^. Early recurrences are related to aggressive tumours, probably associated with micrometastatic disease undetected at operation^30^, ^31^. There is therefore a need to identify more aggressive subtypes of PDAC in order to improve their management.

Ki‐67 is a well known marker of cellular proliferation^7^. Previous experience^3^, ^22^ focusing on PDAC showed that high Ki‐67 expression was associated with poor pathological features, including poor tumour differentiation and presence of lymph node metastasis.

The present study evaluated the prognostic role of Ki‐67 in a series of 170 patients with PDAC and found that patients with a Ki‐67 index above 50 per cent had median DFS and DSS approximately threefold lower than those with a Ki‐67 index of 10 per cent or less (DFS: 8 *versus* 24 months respectively; DSS: 14 *versus* 47 months). In contrast, past reports showed no association between Ki‐67 and overall survival^3^, ^21^, although Ki‐67 index was associated with the risk of recurrence within 1 year after resection^23^.

In the present study a strong association between Ki‐67 index and tumour grade was also found. As expected, the combination of Ki‐67 index above 50 per cent and G3 grade was associated with a greater risk of recurrence and poor survival.

The present results may have clinical implications for patients' prognostic stratification. The Ki‐67 index, as an expression of a more biologically unfavourable disease, might help to discriminate which patients should receive more aggressive adjuvant treatment. Currently, neoadjuvant chemotherapy is recommended for patients with anatomically borderline resectable pancreatic cancer at increased risk of early recurrence^30^, ^32^, ^33^. Preoperative assessment of the Ki‐67 index by EUS–FNA may help to identify patients with marginally resectable tumours based on clinical criteria, who may benefit more from neoadjuvant chemotherapy than upfront surgery, given the high risk of early postoperative recurrence (those with a Ki‐67 index above 50 per cent), although the feasibility of this should be investigated further.

Limitations of this study include its retrospective design and some issues relating to Ki‐67 analysis, including intratumoral and intertumoral heterogeneity^18^, ^21^, ^34^. In addition, the immunohistochemistry protocol may have involved some interobserver variability in determining the percentage of Ki‐67‐positive cells^12^, ^34^. To limit the lack of uniformity and consistency in quantification, several imaging methods have been developed to be used in routine practice^12^, ^18^. However, standardization is needed to enable wider use of the index. Further investigations in larger cohorts are needed to validate these results.

## Supporting information


**Fig. S1** Flow diagram for the study
**Fig. S2** Grading according to Ki‐67 subgroups. Tumour grading distribution (% of G1, G2 and G3) within the three Ki‐67 subgroups (≤ 10%, 11–50%, > 50%).
