# Supplementary material for: Prognostic impact of Ki‐67 proliferative index in resectable pancreatic ductal adenocarcinoma
Source: BJS Open. 2019 May 10;3(5):646–55. doi: 10.1002/bjs5.50175 (PMC6773637; doi:10.1002/bjs5.50175)
Supplement: Supplementary file 1 — Fig. S1 Flow diagram for the study Fig. S2 Grading according to Ki‐67 subgroups. Tumour grading distribution (% of G1, G2 and G3) within the three Ki‐67 subgroups (≤ 10%, 11–50%, > 50%). [file BJS5-3-646-s001.docx]

**BJS5_50175**

**Prognostic impact of Ki-67 proliferative index in resectable pancreatic ductal adenocarcinoma**

**I. Pergolini, S. Crippa, M. Pagnanelli, G. Belfiori, A. Pucci, S. Partelli, C. Rubini, P. Castelli, G. Zamboni and M. Falconi**

**Fig. S1** Flow diagram for the study

**
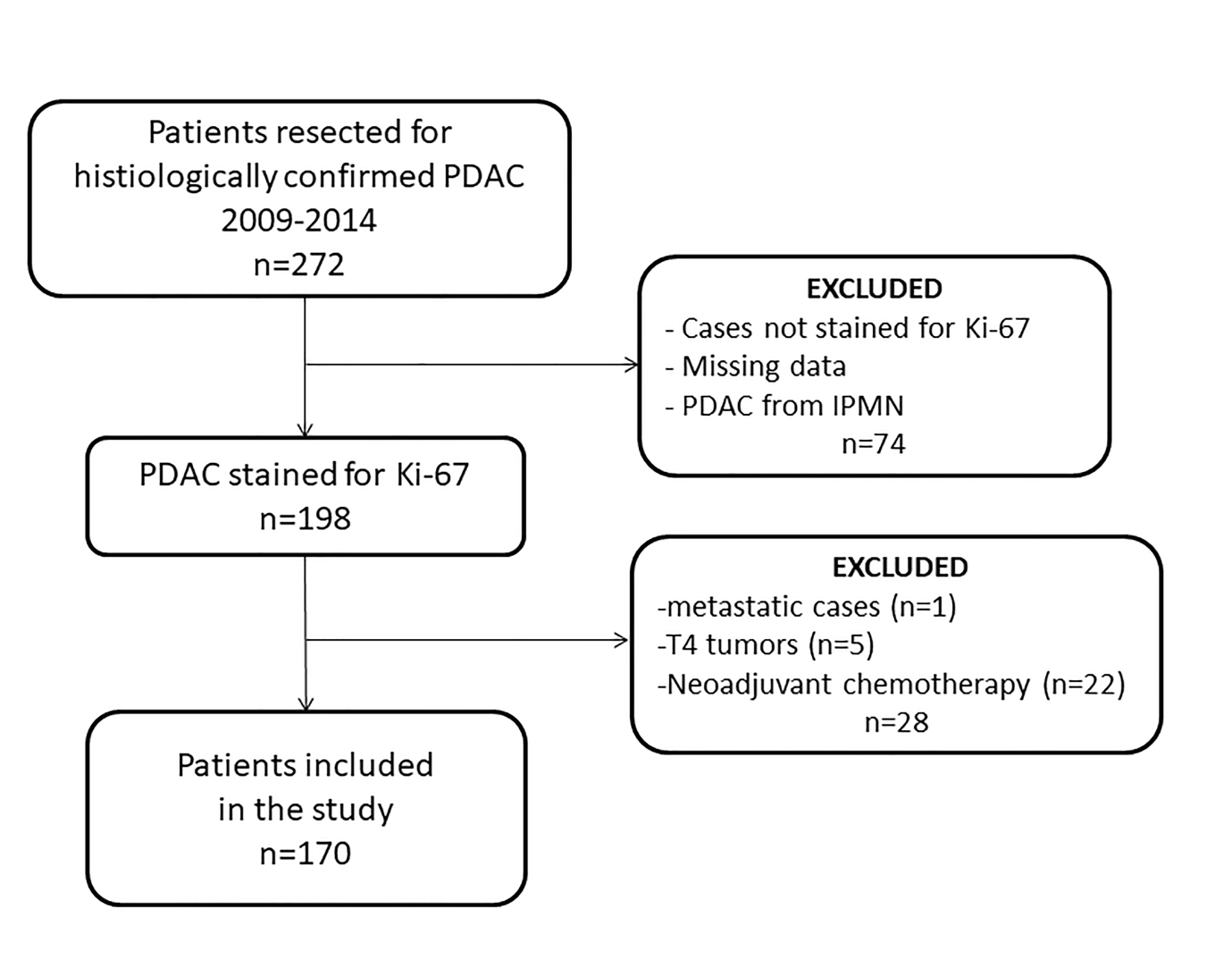
**

**Fig. S2** Grading according to Ki-67 subgroups. Tumour grading distribution (% of G1, G2 and G3) within the three Ki-67 subgroups (≤ 10%, 11–50%, > 50%).
